# Quetiapine-Associated Pancreatitis in a Geriatric Critical Care Patient with Delirium

**DOI:** 10.1155/2012/625954

**Published:** 2012-03-22

**Authors:** Evangelos Potolidis, Charalampos Mandros, Dimitrios Karakitsos, P. M. Kountra

**Affiliations:** ^1^Department of Internal Medicine, General Hospital of Volos, Dafnis 1, 38222 Volos, Greece; ^2^ICU, General State Hospital of Athens, 38222 Athens, Greece

## Abstract

We report the case of a 78-year-old male who developed acute pancreatitis related to quetiapine that was administered for the treatment of delirium. No evidence of hypertriglyceridemia, infection, ischemia, chololithiasis or hypercalcemia could be documented.Clinicians should be alerted when treating critical care patients with antipsychotics, as risks might present and potentially lead to hazardous results.

Quetiapine is a widely used antipsychotic in the treatment of delirium [1]. Although second-generation antipsychotics were associated with pancreatitis, quetiapine-associated pancreatitis is extremely rare [2, 3]. We present a previously healthy 78-years-old Caucasian male who was admitted to the intensive care unit (ICU) following a traffic road accident. The patient was intubated due to a Glasgow Coma Scale of 10. Upon admission, brain computed tomography (CT) scan depicted a small, right-sided subdural hygroma, but otherwise was unrevealing. Abdominal and thoracic CT scans as well as routine biochemistry and blood tests were normal. Weaning from mechanical ventilation was initiated five days after his admission to the ICU as his neurological status upon awakening was normalized. At that time, the patient developed delirium, and he was started on quetiapine 25 mg twice daily, with the dosage being progressively titrated to 50 mg every 12 hours, by the psychiatry consultant. Three days after the initiation of antipsychotic therapy the patient became febrile (38°C), confused, and was complaining of diffuse abdominal pain. Physical examination revealed a mildly distended abdomen, while routine blood tests were normal. Soon after the patient became hemodynamically unstable, and he was reintubated. No evidence of infection could be documented. By-the-bed sonography depicted hypoechoic pancreas without any other pathology. Two days following reintubation, the patient developed leukocytosis (WBC = 16000/lt and 80% neutrophils) and his serum levels of amylase (2210 IU/lt) and lipase (1282 IU/lt) were increased, while his serum levels of alkaline phosphatase, bilirubin, and liver enzymes were normal. No evidence of hypertriglyceridemia, diabetic ketoacidosis or other metabolic abnormality could be documented. Blood, urine, and cerebrospinal fluid cultures were negative. Abdominal CT scans depicted severe acute pancreatitis with peripancreatic fluid, without any other pathology. Quetiapine was discontinued, and the patient was started on broad-spectrum antibiotics and supportive treatment for pancreatitis. Despite all efforts, he developed acute renal failure with ensuing acute respiratory distress syndrome, and he finally expired. Autopsy findings confirmed the diagnosis of pancreatitis.

Pancreatitis could be attributed to various causes such as gallstones, alcohol use, medication toxicity, hypercalcemia and hypertriglyceridemia, trauma from endoscopic retrograde cholangiopancreatography, abdominal trauma, various infections, autoimmune, ischemia, and hereditary causes [4]. Our patient received quetiapine, which is commonly used along with haloperidol, for the treatment of delirium in the ICU [1]. Quetiapine has been associated with the development of hyperglycemia and diabetic ketoacidosis [2, 3]. It remains obscure whether the above metabolic effects of quetiapine are dose dependent [3]. According to published guidelines, patients receiving second-generation antipsychotic drugs should receive baseline screening and ongoing monitoring of plasma glucose and lipid levels [5]. In this case, the use of quetiapine could not be linked to serious metabolic side effects. In the absence of other potential risk factors, we presumed that the development of pancreatitis could be attributed to quetiapine. Further studies are clearly required to investigate this association. Intensivists should be alerted when treating critical care patients with second-generation antipsychotics, as metabolic risks and/or pancreatitis might present and potentially lead to hazardous results.
